# Supplementary material for: Patterns of Intron Gain and Loss in Fungi
Source: PLoS Biol. 2004 Nov 30;2(12):e422. doi: 10.1371/journal.pbio.0020422 (PMC532390; doi:10.1371/journal.pbio.0020422)
Supplement: Table S1 — Also available at http://genes.mit.edu/NielsenEtAl/. (4.3 MB ZIP). [file pbio.0020422.st001.zip › NielsenEtAl/html/157.html]

AN1418.1.NCU04727.1.MG00625.1.FG08398.1


```
 CLUSTAL W (1.82) Multiple Sequence Alignments - Introns Inserted


Sequence 1: MG00625.1	372 aa
Sequence 2: FG08398.1	420 aa
Sequence 3: NCU04727.1	410 aa
Sequence 4: AN1418.1	361 aa
Alignment Length: 436 aa
Number Identitical Residues: 133 aa
Alignment Score (without introns) 6892


MG00625.1 	--MRLIIRDD-DEQ~ASRYVANYIVE~RINHFQPTAAHPFVLGLPTGSSPLGIYRILVEK
NCU04727.1	--MGLIIRDN-AES~ASSYVADYIVD~RINSFSPTRTRPFVLGLPTGSSPLGIYKCLVEK
FG08398.1 	--MRLIIRDD-ETE~ACKYVANYVVE~RINAFHPTPEHPFILGLPTGSSPIGVYNELVRS
AN1418.1  	MYVASRIRPCGRAD0ASEYIADYIIS1RIKAYKPSESNPFVLGLPTGSSPEIIYKTLVRR
          	  :   **  .  . *. *:*:*::. **: : *:  .**:*********  :*. **. 

MG00625.1 	YKAGE0ISFENVVTFNM~DEYIGIPRDHPESYHSFMWKHFFSHVNVNPANVHILDGNAPN
NCU04727.1	YKAGL~VSFENVITFNM0DEYVSLPPTHPQSYASFMHDNFFSHVNIPPQNTNLLNGLAPD
FG08398.1 	YKAGQ0VSFENVVTFNM~DEYVGLPRDDPNSYHSFMWKHFFSHVNIHPSNVHILNGNAAS
AN1418.1  	YKAGD~ISFRHVVTFNM0DEYVGLPRDHPESYHSFMYKHFFSHVDIPPQNINILDGNATD
          	****  :**.:*:**** ***:.:*  .*:** *** .:*****:: * * ::*:* *..

MG00625.1 	LEAECVAYEESIRRAGGIDLFLAGIGEDGHIAFNEPGSSLASRTRVKTLAYDTILANSRF
NCU04727.1	LAAECSRYEAKIAAAGGIDLFLAGLGEDGHLAFNEPGSSLASRTRVVALAEDTILANSRF
FG08398.1 	PEAECDAYEEAIKAVGGIDLFLAGIGEDGHIAFNEPGSSLASRTRVKTLAYDTILSNSRF
AN1418.1  	LAAECASFEARIARYGGIELFLGGVGSDGHIAFNEPGSSLNSRTRVKTLAYDTILANSRF
          	  ***  :*  *   ***:***.*:*.***:********* ***** :** ****:****

MG00625.1 	FGNDMEKVPKMALTVGVQTVLEAREVVVIILGARKALALQRCLEQGVNHMWTLSSLQLHP
NCU04727.1	FDDDVNKVPQLALTVGVKTVLEAREVLMIVLGAKKARALKKCVEEGVSSMWTGSALQMHE
FG08398.1 	FDNDVSKVPRMALTVGVQTVLEAKEVVVIILGARKALALQKCVEQGVNHMWSLSCLQMHP
AN1418.1  	FNNDMAQVPRMALTVGIRTIMEAREVVIVATGAHKALAVKEGLEGGVNHMWTISSLQLHQ
          	*.:*: :**::*****::*::**:**:::  **:** *::. :* **. **: *.**:* 

MG00625.1 	HPMIVCDEDATLELQ~VKTVK0YFKSIEQ--VAKTQGFEQILPSKMRTGTVPIAPRVVLD
NCU04727.1	RATVICDEEAAGELK~WKTVK0YFKSVERREFGHSHGSDQSLPIRKGLGTPPTKLKTAAG
FG08398.1 	HPMIVVDEDATLELQ0-----~YFKSIEK--VAREQGFEQILPSKVRTGNVAIPETKIH-
AN1418.1  	HPLIVCDRDATLELK~VKTVR~YFESIEQ---AGTDARTQGPPLVYRPRTYVPAPMTVNK
          	:. :: *.:*: **:  .:   **:*:*:   .  ..  *  *      .          

MG00625.1 	EEVQAPTVHAPKPVTSSLLHALTPAAEYPVRSVSPDLVPDRMGSRIPGDSEAAFHRRLTP
NCU04727.1	PLTPESTPKATATRSSSSPIS--PLAQTVPILLTTNVKPAQSSGYIA--LEQKILAQPVP
FG08398.1 	-RTQSPVIIAPEPIASHLLRA-TPMGDYSMRTPSPDLLPDRMASRIP---EPNLNRRLTP
AN1418.1  	SNGELTPIATPEKTSKDLRIN----TDLTRSFEDDELTPDSMSSRMVDSAVGGLDATLKG
          	     .   :.   :.          :        :: *   .. : ..    :      

MG00625.1 	NPEQQ-DLHKQKL----SHAFVSTQLVA--------------------------------
NCU04727.1	KSHGLLGLLGTSS---YGQTSDGDSEAESEYDLKP-----------DRMASRLTDPVFAA
FG08398.1 	NLEVQTDVPKTKVDMIDSAVAMSPEQVASDLLLQPPMKKTMRSPSPDLIPDRMASRIPEP
AN1418.1  	------DLMFDRMG--------ARMEAH--------------------------------
          	      .:     .        .   .                                 

MG00625.1 	----------------------
NCU04727.1	EALRRLTPNPETGKMLG-----
FG08398.1 	SLNGRLTPSPELKNRMNTVGAH
AN1418.1  	----------------------
          	
```
